# Emerging roles of tsRNAs in programmed cell death and disease therapeutics: challenges, opportunities, and future directions

**DOI:** 10.1016/j.ncrna.2025.07.003

**Published:** 2025-07-11

**Authors:** Zhe Li, Bo Zhang, Yanru Pan, Qiuyan Weng, Kefeng Hu

**Affiliations:** a Department of Gastroenterology, The First Affiliated Hospital of Ningbo University, 315010, Ningbo, China; b Ningbo Key Laboratory of Translational Medicine Research on Gastroenterology and Hepatology, 315010, Ningbo, China

**Keywords:** tsRNAs (tRNA-derived small RNAs), Programmed cell death (PCD), Diagnostic biomarkers, Therapeutic targets, Clinical translation

## Abstract

Programmed cell death (PCD), which includes various forms such as apoptosis, autophagy, necroptosis, pyroptosis, and ferroptosis, plays a pivotal role in disease pathogenesis and progression. tRNA-derived small RNAs (tsRNAs) have emerged as crucial regulators of these processes, influencing cellular fate and disease outcomes. Research has revealed diverse expression profiles of tsRNAs across various diseases, emphasizing their roles in modulating PCD pathways and their potential value in diagnosis and treatment. Specific tsRNAs can either promote or inhibit apoptosis; for example, tsRNA-3043a promotes ovarian granulosa cell apoptosis in premature ovarian insufficiency, whereas tsRNA-04002 prevents apoptosis in nucleus pulposus cells to delay intervertebral disc degeneration. Furthermore, tsRNAs serve as potential biomarkers for early disease detection, with emerging detection technologies enhancing their clinical utility. Therapeutically, tsRNA-targeted strategies, such as RNA interference and exosome-based drug delivery, offer new avenues for modulating PCD in diseases such as cancer, cardiovascular disorders, and neurodegenerative diseases. Despite challenges in understanding tsRNA biogenesis and functional diversity, their roles in regulating PCD highlight their strong potential in advancing disease diagnostics, treatment strategies, and personalized medicine.

## 1 Introduction

Programmed cell death (PCD) is a genetically regulated process essential for maintaining cellular homeostasi. It encompasses several distinct forms, including apoptosis, autophagy, necroptosis, pyroptosis, and ferroptosi [1,2]. Dysregulating PCD pathways has been implicated in the pathogenesis of various diseases, such as cancer, cardiovascular disorders, and neurodegenerative conditions [3,4]. In recent years, tRNA-derived small RNAs (tsRNAs), a novel class of non-coding RNAs (ncRNAs) produced by the cleavage of tRNAs under stress or pathological conditions, have emerged as critical regulators of PCD and disease progression [5].

tsRNAs are classified into distinct subtypes, such as tRF-1s, tRF-5s and tiRNAs, based on their biogenesis and cleavage sites. They play diverse roles in regulation of gene expression, translation modulation, and epigenetic reprogramming [6]. For instance, tsRNA-3043a promotes apoptosis in ovarian granulosa cells by targeting *FLT1*, thereby exacerbating premature ovarian insufficiency [7], In contrast, tsRNA-04002 inhibits apoptosis in nucleus pulposus cells by suppressing *PRKCA*, thus delaying intervertebral disc degeneration [8]. Such disease-specific tsRNA expression profiles highlight the potential of tsRNAs as both diagnostic biomarkers and therapeutic targets. In acute coronary syndrome (ACS), tRF-Gly-GCC-06 correlates with Gensini scores and independently predicts ACS risk [9]. Similarly, tsRNA-12391 promotes mitophagy and alleviates osteoarthritis by upregulating *PINK1* and *LC3* [10]. These findings underscore the dual roles of tsRNAs in promoting or mitigating disease progression through PCD modulation.

Emerging detection technologies such as CRISPR-Cas–based systems and catalytic hairpin assembly (CHA) have enabled the sensitive detection of tsRNAs (e.g., with limits of 0.215 fM for miRNA-155) [11,12], paving the way for their clinical translation. Furthermore, tsRNA-targeted therapies, such as the self-delivering RNAi drug PH-762, which silences *PD-1*, have demonstrated remarkable antitumor effects by enhancing T-cell immunity and inducing systemic abscopal responses [13]. Despite these advances, controversies persist regarding tsRNA biogenesis mechanisms and context-dependent functionalities. For instance, tsRNA-26576 has demonstrated both oncogenic and tumor-suppressive effects across different breast cancer subtypes [14], thereby emphasizing the need for precision in therapeutic applications.

This review summarized current knowledge on tsRNA-mediated regulation of PCD, explored their diagnostic and therapeutic implications, and discussed key challenges in translating these findings into clinical practice. Future research integrating multiomics approaches and advanced delivery platforms, such as nanoparticle systems, holds great promise for unlocking the full potential of tsRNAs in personalized medicine.

## 2 tsRNAs and PCD: mechanisms, diagnostic value, and therapeutic potential

### 2.1 Biological characteristics and classification of tsRNAs

tsRNAs are small RNAs derived from tRNAs, which have gained significant attention in recent years [15,16]. They are not produced randomly but are instead generated under specific conditions through the cleavage of precursor or mature tRNAs by specific nucleases, such as Elac Ribonuclease Z 2 (ELAC2)/RNase Z, RNase L, Dicer, and angiogenin (ANG) [15]. tsRNAs can be classified into several types based on the location of their origin on the parental tRNA: tRF-1s, tRF-3s, tRF-5s, tsRNAs, and tRF-2s/i-tRFs [15]. Another widely used classification, which is based on differences in cleavage sites, divides them into tRNA fragments (tRFs) and tRNA-derived stress-induced small RNAs (tiRNAs) [5,16].

tsRNAs perform various biological functions, including gene expression regulation, inhibition of apoptosis, translational control, and epigenetic modification [6,17]. Their abnormal expression has been observed in various diseases, suggesting their significant role in disease onset and progression. These biological characteristics establish tsRNAs as key molecular regulators of PCD. In the following sections, we discuss in detail the mechanisms through which tsRNAs influence apoptosis, pyroptosis, and ferroptosis.

### 2.2 Basic mechanisms of PCD

PCD is a gene-regulated process that occurs under physiological or pathological conditions and is vital for maintaining the internal stability of the organism [1]. The main forms of PCD include apoptosis, autophagy, and programmed necrosis. Apoptosis is the most common type of PCD, triggered by the intrinsic (mitochondrial) or extrinsic (death receptor) pathways, which activate a caspase cascade leading to cell death [3]. Autophagy involves the formation of autophagosomes, which encapsulate and degrade damaged organelles and proteins within the cell. It plays a dual role in promoting cell survival and inducing cell death. Moderate autophagy helps maintain cellular homeostasis, whereas excessive autophagy can lead to cell death [18]. Programmed necrosis, such as necroptosis, is mediated by receptor-interacting protein kinases (RIPKs) and plays a role in inflammatory responses and tissue damage [2]. Additionally, other forms of PCD, such as pyroptosis and ferroptosis, are characterized by distinct molecular mechanisms and play specific roles in various physiological and pathological processes.

### 2.3 Role of tsRNAs in apoptosis

tsRNAs play a crucial role in apoptosis. Studies have shown that tsRNA-3043a can enhance the apoptosis and senescence of ovarian granulosa cells by targeting FLT1, thereby promoting the development of premature ovarian insufficiency [7]. In this study, cell and mouse models of premature ovarian insufficiency were established. tsRNA-3043a was upregulated in the ovarian tissues of the model mice. Its mimic inhibited the proliferation of ovarian granulosa cells and promoted apoptosis, lipid accumulation, and cellular senescence, whereas its inhibition exerted opposite effects.

**Fig. 1:**
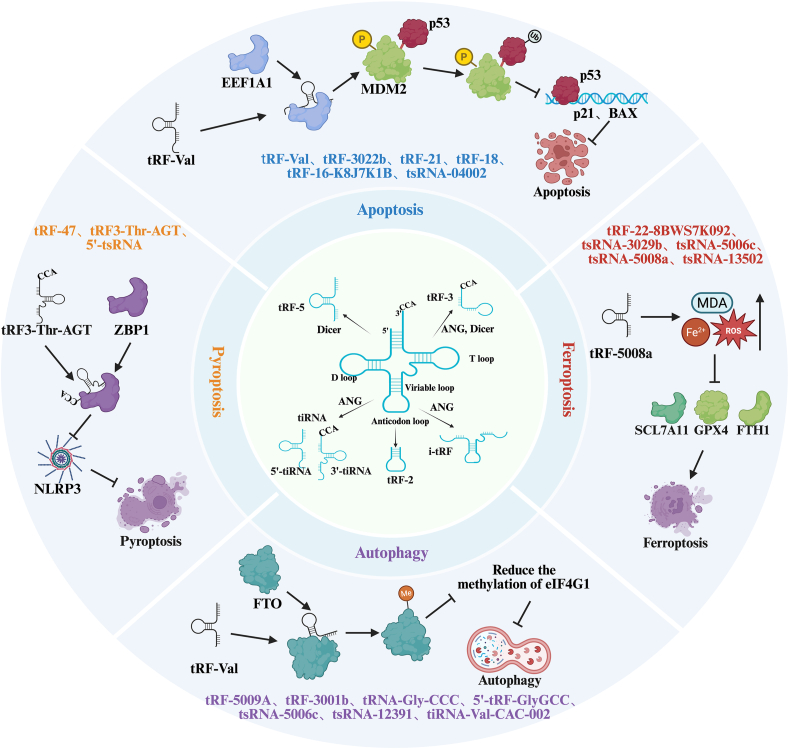
Schematic representation of tsRNA-mediated regulation in various forms of programmed cell death.

Conversely, tsRNA-04002 inhibits apoptosis in nucleus pulposus cells by targeting *PRKCA*, thereby alleviating intervertebral disc degeneration [8]. Small RNA sequencing of nucleus pulposus tissues from different patients with disc degeneration revealed that tsRNA-04002 expression was significantly downregulated in the patient tissues. Experimental overexpression of this tsRNA inhibited the expression of inflammatory factors in nucleus pulposus cells, increased *COL2A1* expression, and suppressed apoptosis [8]. These findings indicated that tsRNAs regulated apoptosis by modulating gene expression to influence the process of apoptosis, thereby playing a role in the onset and progression of various diseases. As illustrated in Fig. 1, key tsRNAs, such as tsRNA-3043a (targeting FLT1) and tsRNA-04002 (targeting PRKCA), modulate apoptosis through distinct molecular pathways. For instance, tsRNA-3043a enhances apoptosis by activating the mitochondrial pathway, whereas tsRNA-04002 suppresses apoptosis by downregulating inflammatory signaling. This comprehensive regulatory network highlights the multifaceted roles of tsRNAs in PCD.

### 2.4 Expression profiles of tsRNAs in different diseases

tsRNAs exhibit disease-specific expression profiles in various conditions, making them attractive candidates as biomarkers for diagnosis and prognosis [9,19]. In patients with ACS, high-throughput small RNA sequencing of peripheral blood mononuclear cells revealed unique tsRNA expression patterns. Specifically, tRF-Gly-GCC-06 was significantly upregulated in patients with unstable angina and acute myocardial infarction. This tsRNA was positively correlated with Gensini scoresand found to be independently associated with increased ACS risk [9]. The study sequenced 24 patients with ACS and 12 healthy controls, with validation conducted in two independent case–control cohorts.

Additionally, differential tsRNA expression was observed in the peripheral blood of monozygotic twins, suggesting that tsRNAs may serve as biomarkers for distinguishing between twins [19]. The study analyzed the peripheral blood tsRNAs of four pairs of adult monozygotic twins, identifying 8795 expressed tsRNAs using PANDORA-seq technology [20]. After screening, 10 differentially expressed tsRNAs capable of distinguishing between twins were validated using qRT-PCR and ddPCR.

### 2.5 Correlation between tsRNAs and PCD

The advancement in research on the relationship between tsRNAs and PCD has provided new perspectives for understanding disease mechanisms. Studies have demonstrated the pivotal roles of specific tsRNAs in different types of PCD such as apoptosis, autophagy, and ferroptosis. For instance, tsRNA-3043a promotes apoptosis and senescence in ovarian granulosa cells, whereas tsRNA-04002 inhibits apoptosis in nucleus pulposus cells. These studies highlight the diverse and complex roles of tsRNAs in regulating cell death in various diseases [4].

Table 1 provides an overview of the roles of various tsRNAs in different PCD pathways, highlighting their molecular targets and impact on diseases such as osteoarthritis, cancer, and cardiovascular conditions. Understanding these functions can aid in developing targeted therapeutic strategies focused on specific tsRNAs. Using the MSBB RNA-seq dataset and methods such as weighted gene co-expression network construction, researchers have identified RNA modification-related PCD genes that can help classify patients into subgroups with distinct clinical characteristics. Based on these findings, a risk score model was developed for disease diagnosis and progression assessment.

**Table 1:** The role of different tsRNAs in programmed cell death pathways and their impact on disease progression.

Type	tsRNA Name	Target Pathway/Target	Disease Type	Impact on disease	Model/Cell type	Ref
Autophagy	tRF-5009A	mTOR pathway	Osteoarthritis	Inhibits autophagy, worsens degeneration	Cartilage specimens	[22]
	tRF-3001b	Inhibits key autophagy node	Non-alcoholic fatty liver disease	Promotes lipid buildup, worsens disease	C57BL/6J mice	[23]
	tRNA-Gly-CCC	Downregulates RNAP III signaling	Chondrosarcoma	Induces autophagy-dependent cell cycle arrest	Chondrosarcoma cells	[24]
	5′-tRF-GlyGCC	Inhibits eIF4G1 methylation	Breast cancer	Promotes metastasis by blocking autophagy	Breast cancer cells	[25]
	tsRNA-5006c	Regulate mitochondrial autophagy	Aortic valve calcification	Promotes osteogenic differentiation of cells	Macrophages	[26]
	tsRNA-12391	ATAD3A/PINK1-mediated mitophagy pathway	Osteoarthritis (OA)	Promotes mitophagy, alleviates cartilage degeneration	Mesenchymal stem cells	[10]
	tiRNA-Val-CAC-002	ITGB3/PI3K/AKT autophagy pathway	Oral Submucous Fibrosis (OSF)	Promotes fibroblast activation, worsens fibrosis	Fibroblasts	[27]
Ferroptosis	tRF-22-8BWS7K092	Activates Hippo pathway	Acute lung injury	Induces ferroptosis, worsens inflammation	C57BL/6 mice	[28]
	tsRNA-3029b	Inhibits key ferroptosis pathway	Major Depressive Disorder	Reduces ferroptosis, alleviates depression	C57BL/6 mice	[29]
	tsRNA-5006c	SLC7A11/GPX4^a^ ferroptosis axis	Perioperative Neurocognitive Disorders (PND)	Suppresses ferroptosis, improves cognitive function	C57BL/6 mice	[30]
	tsRNA-5008a	SLC7A11/GPX4 ferroptosis axis	Atrial Fibrillation (AF)	Promotes atrial fibrosis, electrical remodeling	C57BL/6 mice	[31]
	tsRNA-13502	SLC7A11/GPX4 ferroptosis axis	AF	Promotes liver inflammation via pyroptosis	Non-small cell lung cancer cells	[32]
Pyroptosis	tRF-47	NLRP3 inflammasome regulation	Non-alcoholic steatohepatitis (NASH)	Reduces pyroptosis, alleviates tissue damage	C57BL/6 mice	[33]
	tRF3-Thr-AGT	Inhibits ZBP1/NLRP3 pathway	Acute pancreatitis	Blocking interaction mitigates organ damage	Rat pancreatic acinar cells	[34]
	5′-tsRNA	DDX3X-NLRP3 Interaction	Septic Shock, Type-2 Diabetes	Promotes mitophagy, alleviates cartilage degeneration	C57BL/6J mice	[35]
Apoptosis	tRF-Val	Targets EEF1A1	Gastric cancer	Inhibits apoptosis, promotes proliferation	Gastric cancer cells	[36]
	tRF-3022b	Regulates cytokine signaling	Colorectal cancer	Inhibits apoptosis, promotes macrophage polarization	Colorectal cancer cells	[37]
	tRF-21	Regulates inflammatory pathway	Pancreatic ductal adenocarcinoma	Inhibits progression via pro-apoptotic effect	Pancreatic ductal adenocarcinoma cells	[38]
	tRF-18	Inhibits KIF1B expression	Papillary thyroid carcinoma	Blocks mitochondrial apoptosis, promotes growth	Papillary thyroid cancer cells	[39]
	tRF-16-K8J7K1B	Drug resistance-related pathway	Breast cancer	Reduces apoptosis, enhances tamoxifen resistance	Breast cancer cells	[40]
	tsRNA-04002	Targets PRKCA	Intervertebral disc degeneration	Inhibits apoptosis, delays degeneration process	Nucleus pulposus cells	[8]

a GPX4: Glutathione peroxidase 4.

In cancer research, metal-dependent PCD-related long noncoding RNAs (lncRNAs) have been shown to influence the prognosis of gastric cancer. tsRNAs may be linked to these lncRNAs and PCD [21]. A prognostic signature model consisting of 12 lncRNAs was developed by analyzing data from patients with gastric cancer, demonstrating strong predictive accuracy for overall survival in these patients. Additionally, the study explored the potential of using traditional Chinese medicines and plant extracts to induce metal-dependent PCD, suggesting that tsRNAs may contribute to cancer development through similar mechanisms. Fig. 1 summarizes the key tsRNAs, their molecular targets, and associated signaling pathways involved in apoptosis, autophagy, ferroptosis, and pyroptosis, aiming to provide a more intuitive overview of the complex regulatory roles of tsRNAs across different types of PCD.

### 2.6 Interaction between tsRNAs and autophagy

There appears to be a strong interaction between tsRNAs and autophagy. Studies have shown that exosomes derived from adipose-derived mesenchymal stem cells, modified by tsRNA-12391, can alleviate cartilage degeneration in osteoarthritis (OA) by enhancing mitophagy [10]. In this study, small RNA sequencing identified OA-related tsRNAs; tsRNA-12391 was found to be downregulated in the OA group. Mitophagy was promoted after introducing a tsRNA-12391 mimic into cells, as evidenced by the upregulation of PINK1 and LC3 and increased co-localization of Mito-Tracker Green and PINK1. Additionally, this process enhanced the chondrogenic differentiation of cartilage cells. These findings suggest that tsRNA-12391 may regulate mitophagy-related pathways by interacting with associated proteins, thereby influencing the fate of chondrocytes.

Other studies have also demonstrated that some RNA-binding proteins can affect autophagy by regulating the expression of autophagy-related genes. As illustrated in Fig. 1, tsRNAs such as tRF-5009A and tRNA-Gly-CCC are pivotal in modulating autophagy-related pathways. For example, tRF-5009A inhibits the mTOR pathway and worsens cartilage degeneration, whereas tRNA-Gly-CCC downregulates RNAP III signaling to induce autophagy-dependent cell cycle arrest. These findings suggest that tsRNAs may regulate autophagy through similar mechanisms, although their specific modes of action and targets require further investigation.

### 2.7 Role of tsRNAs in PCD of tumor cells

tsRNAs play a vital role in the PCD of tumor cells, influencing tumor development and progression. In non-small-cell lung cancer, vitamin D can induce mitochondrial dysfunction and inhibit tumor progression by upregulating tsRNA-07804, which targets CRKL [41]. The study showed that tsRNA-07804 was significantly upregulated after vitamin D treatment in H1299 cells. Functional experiments revealed that vitamin D-mediated regulation of tsRNA-07804 led to mitochondrial dysfunction, suppressed cell proliferation, migration, and invasion, and promoted apoptosis.

In breast cancer, tsRNA-26576 is significantly upregulated and may promote cell proliferation and migration while inhibiting apoptosis. This tsRNA has the potential to serve as both a therapeutic target and a prognostic biomarker for breast cancer [14]. Small RNA sequencing of tissues of patients with breast cancer revealed that tsRNA-26576 was significantly upregulated in cancer tissues. Further experiments confirmed its role in influencing the biological behavior of breast cancer cells.

These findings suggest that tsRNAs play an essential role in regulating PCD in tumor cells and may offer new therapeutic targets and strategies for cancer treatment.

### 2.8 Detection technologies for tsRNAs as biomarkers

PCD plays a crucial role in the pathogenesis of various diseases, including cardiovascular, neurodegenerative, and malignant conditions. In cardiovascular diseases, abnormal PCD, such as apoptosis, necrosis, pyroptosis, ferroptosis, and autophagy, contributes to myocardial injury and dysfunction, especially in acute myocardial infarction [42]. In neurological disorders such as Alzheimer's disease (AD), neuronal apoptosis and necrosis are associated with progressive cognitive decline [4]. In cancer, the resistance to apoptosis in tumor cells and dysregulated death of immune cells in the tumor microenvironment drive tumor progression and immune evasion [43]. These disease-specific PCD signatures underscore the need for sensitive and specific biomarkers, an area where tsRNAs have shown great promise.

Accurate detection of tsRNAs is essential for their application as biomarkers in disease diagnosis. Several advanced detection technologies have been developed, including next-generation sequencing (NGS), CRISPR-Cas-based systems, and CHA. These techniques offer high sensitivity and allow for comprehensive analysis of tsRNA profiles, which is crucial for understanding disease mechanisms and improving diagnostic accuracy. For instance, CRISPR-Cas systems and NGS enable the profiling of tsRNA expression in diseases such as cancer and cardiovascular conditions [11].

Table 2 summarizes these advanced detection technologies and their applications. These methods have proven effective in profiling tsRNA expression patterns across various diseases, including cancer, cardiovascular disorders, and neurological conditions, underscoring their potential in disease-specific detection and targeted therapeutic applications.

**Table 2:** Advanced Technologies for tsRNA Detection and Targeting in Disease Diagnosis and Therapy.

Technology	Description	Associated tsRNAs	Applications
**Next-Generation Sequencing**	High-throughput sequencing for comprehensive profiling of tsRNAs.	tRF-5009A, tRF-3001b, tRNA-Gly-CCC	Used for profiling tsRNA expression patterns in various diseases (e.g., cancer, cardiovascular diseases).
**CRISPR-Cas Technology**	CRISPR/Cas-based systems to manipulate tsRNA expression.	tsRNA-3043a, tsRNA-04002	Used in gene editing for regulating tsRNA expression in disease models.
**Catalytic Hairpin Assembly**	Isothermal amplification technique to detect specific tsRNAs at low concentrations.	tRF-22-8BWS7K092, tsRNA-3029b	Point-of-care detection of disease-specific tsRNAs, such as in pancreatic cancer.
**Surface-Enhanced Raman Spectroscopy**	Biosensor technology for ultra-sensitive detection of small RNAs like tsRNAs.	miRNA-21, miRNA-155 (closely related to tsRNAs)	Sensitive detection of biomarkers in breast cancer, improving diagnostic precision.
**RNA Interference**	Use of small interfering RNAs (siRNAs) to silence or modulate tsRNA activity.	tsRNA-26576, tsRNA-5008a	Used to inhibit specific tsRNAs in cancer therapy to reduce tumor progression.
**Exosome-based Delivery**	Exosome-mediated delivery of tsRNA mimics or inhibitors to target cells.	tsRNA-12391	Delivery of tsRNAs to alleviate cartilage degeneration in osteoarthritis.
**Biosensors (e.g., electrochemical)**	Electrochemical sensors combined with amplification technologies for detecting tsRNAs.	tRF-3022b, tsRNA-3029b	Biosensors for detecting tsRNAs in colorectal cancer for early-stage diagnosis.
**RNA-Seq Combined with Machine Learning**	Integration of RNA-Seq data with machine learning algorithms for predicting tsRNA expression profiles.	tRF-Gly-GCC-06, tRNA-Val	Predicts disease progression, such as in acute coronary syndrome, by analyzing tsRNA profiles.
**Nanoparticle-based Delivery**	Nanoparticles designed for targeted delivery of tsRNA mimics or inhibitors.	tsRNA-5006c, tsRNA-5008a	Used in cancer therapy, targeting specific tsRNAs to promote tumor cell apoptosis.

Additionally, biosensors utilizing surface-enhanced Raman spectroscopy (SERS) combined with CHA have been developed for the ultra-sensitive detection of miRNA-21 and miRNA-155 in breast cancer serum. These biosensors demonstrate detection limits as low as 0.398 and 0.215 fM, respectively, with a dynamic range from 1 fM to 10 nM. They can distinguish between patients with breast cancer and healthy controls with 100 % accuracy and may also aid in molecular subtyping and prognosis assessment [12]. The development of such technologies provides robust support for the clinical application of tsRNAs as diagnostic biomarkers.

### 2.9 Development of diagnostic reagents for PCD-related tsRNAs

Progress has been made in developing diagnostic reagents targeting PCD-related tsRNAs. For example, soluble PCD protein 1 (sPD-1) and soluble PCD-ligand 1 (sPD-L1) have been identified as potential biomarkers for the diagnosis and prognosis of glioma. Researchers used ELISA kits to detect sPD-1 and sPD-L1 levels in serum, revealing the highest preoperative sPD-1 levels in patients with glioma. The diagnostic performance was notable, with sPD-1 (AUC = 0.762) and sPD-L1 (AUC = 0.718) demonstrating similar efficacy. Additionally, high serum levels of sPD-1 (>11.14 pg/mL) and sPD-L1 (>63.03 pg/mL) were linked to shorter progression-free survival in patients with glioma [44].

Although this study focused on sPD-1 and sPD-L1, it provides valuable insights for developing diagnostic reagents based on tsRNAs related to PCD.

In neurodegenerative diseases such as AD, PCD is also regarded as a profound research direction. Investigating PCD-related biomarkers in AD may help scientists uncover the mechanisms underlying neuronal loss, thereby facilitating the development of novel therapeutic approaches. Leveraging bioinformatics, researchers have identified several PCD-associated AD biomarkers and proposed potential treatment options through drug repositioning studies [45].

Future studies may focus on developing diagnostic reagents combining tsRNAs with PCD-related proteins to improve diagnostic accuracy and reliability.

### 2.10 Clinical applications of tsRNAs: diagnosis and therapy

tsRNAs have significant potential for application in early disease diagnosis. A model has been developed to predict malignant lung nodules by establishing a nomogram based on the diagnostic features of five circulating tsRNAs and CT imaging data. This model displayed high accuracy in both internal validation (*n* = 83, AUC = 0.930, sensitivity of 100.0 %, specificity of 73.8 %) and external validation (*n* = 66, AUC = 0.943, sensitivity of 100.0 %, specificity of 86.8 %) cohorts, and also performed well in distinguishing invasive malignant lesions from noninvasive ones [46].

In liver cancer diagnosis, the serum mitochondrial tsRNA tRF-Gln-TTG-006 has been identified as a novel biomarker. It can differentiate patients with liver cancer from healthy controls in an early stage, with a sensitivity of 79.0 % and specificity of 74.8 % across two independent cohorts (155 healthy controls and 153 patients with liver cancer) [47].

**Fig. 2:**
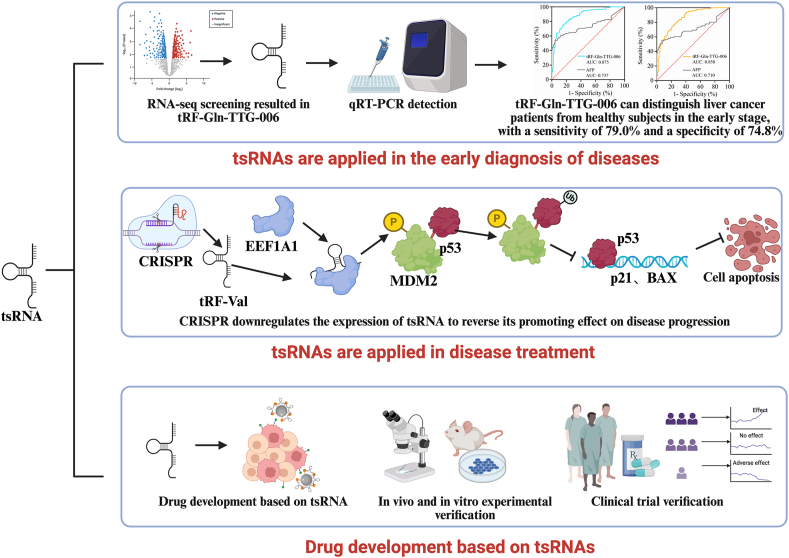
Clinical applications and future directions of tsRNAs in disease diagnosis, mechanism exploration, and therapeutic development.

These findings suggest that tsRNAs offer promising strategies for early disease detection and diagnosis, potentially improving early detection and treatment outcomes. Fig. 2 outlines the clinical applications of tsRNAs, including their use as diagnostic tools (e.g., NGS and SERS biosensors) and therapeutic strategies (e.g., RNAi-based therapies and exosome delivery systems). Integrating tsRNA profiles with machine learning algorithms has enabled the development of predictive models for diseases such as ACS, achieving high diagnostic accuracy (AUC > 0.93). These advancements align with the future directions highlighted in Fig. 2, emphasizing the potential of tsRNAs in precision medicine.

In breast cancer, elevated tsRNA expression is associated with poor survival outcomes, whereas reduced expression correlates with a better prognosis [48]. Similarly, the altered expression of tsRNAs in gastric cancer has been used for diagnosis and prediction of treatment responses [49]. In neurogenetic disorders, tsRNAs exhibit significant expression changes, suggesting their potential applications in the diagnosis and treatment of these diseases [50]. Furthermore, aberrant tsRNA expression in digestive system disorders highlights their potential as diagnostic and prognostic biomarkers. Urinary exosomal tsRNAs have also been investigated for diagnosing systemic lupus erythematosus nephritis. Studies indicate that these tsRNAs can serve as noninvasive biomarkers for effective diagnosis and prediction of lupus nephritis [51].

tsRNA-targeted therapy is an emerging therapeutic strategy with strong potential for application. For example, tsRNA-3043a has been found to promote disease progression in early-onset ovarian insufficiency by targeting FLT1. This suggests that targeting tsRNA-3043a and its associated pathways can lead to the development of new drugs aimed at slowing or reversing the progression of early-onset ovarian insufficiency [7]. In cancer treatment, RNA interference (RNAi) technology offers a method for tsRNA-targeted therapy using small interfering RNA (siRNA) to silence specific gene expression [52]. siRNAs can be designed to target tsRNAs involved in PCD in tumor cells. The key cellular behaviors, such as proliferation and apoptosis, can be regulated by modulating the function of these tsRNAs, either inhibiting or enhancing their function. Although several siRNA-based drugs have already entered clinical trials, challenges such as effective drug delivery and potential immune responses still remain. Nevertheless, ongoing technological advancements provide the hope that tsRNA-targeted therapies can lead to significant breakthroughs in the treatment of various diseases.

### 2.11 Drug development for regulating PCD

The development of drugs that regulate PCD has become a current research hotspot. For example, the PD-1/PD-L1 pathway plays a crucial role in tumor immune evasion, and drugs that block this pathway have become essential tools in cancer treatment [53]. However, patient responses to PD-1/PD-L1 inhibitors can vary significantly, largely due to differences in how PD-L1 expression is regulated in tumor cells [54]. Studies have found that DNA damage signaling can influence PD-L1 expression. Therefore, a deeper understanding of the regulatory mechanisms of PD-L1 expression can help develop more effective PD-1/PD-L1-targeted drugs. Moreover, drugs targeting other molecules involved in PCD, such as key molecules regulating apoptosis, autophagy, and necroptosis, can help in developing more precise disease treatments.

In the context of drug development, tsRNAs have emerged as novel regulatory molecules with the potential to modulate PCD. tsRNAs can regulate cellular survival and death by influencing gene expression and signaling pathways. Therefore, investigating the mechanistic role of tsRNAs in PCD and exploring its potential as a drug target may provide a profound theoretical foundation and practical prospects for developing novel therapeutic strategies. Building on the mechanistic role of SPK-1 in PCD, future studies could examine the interaction between tsRNA and SR protein kinases, as well as their synergistic effects in cell death regulation. This may help unravel the more intricate regulatory networks involved in cell death and offer innovative approaches for treating related diseases [55].

### 2.12 Application of tsRNAs in immunotherapy

tsRNAs hold potential in immunotherapy. For example, in preclinical studies, a self-delivering RNAi immune therapy drug, PH-762, designed to silence PD-1, showed promising antitumor effects [13]. PH-762 was quickly internalized by human T cells, silenced PD-1 mRNA, and reduced PD-1 surface protein levels, thus enhancing IFN-γ and CXCL10 secretion upon TCR stimulation. In *in vivo* experiments, the local injection of mPH-762 (the mouse equivalent of PH-762) not only significantly inhibited local tumors but also exerted distant effects on untreated distal tumors by inducing systemic antitumor immunity. These findings highlight the potential of tsRNA-based immunotherapy strategies in effective cancer treatment.

Recent studies have shown a significant role of tsRNAs in the tumor immune microenvironment. They can influence tumor immune evasion and resistance to immunotherapy by modulating the function and differentiation of immune cells [56]. For instance, tsRNAs can regulate the efficacy of immune checkpoint blockade therapy by interacting with immune checkpoint molecules, thereby enhancing antitumor immune responses [57]. In cancer immunotherapy, the regulatory role of tsRNAs extends beyond tumor cells themselves to include the functional modulation of tumor-associated immune cells. Research has revealed that tsRNAs can impact the recruitment, activation, and polarization of immune cells, thereby influencing tumor cell proliferation and metastasis [58]. For example, tsRNAs may help regulate antitumor immune responses by affecting the differentiation and maturation of immune cells [59].

In summary, tsRNAs hold great potential in tumor immunotherapy. A deeper understanding of their regulatory roles within the tumor immune microenvironment can provide a theoretical foundation and practical guidance for developing novel immunotherapeutic strategies [60]. Future research should focus on elucidating the specific mechanisms of tsRNAs in tumor immune regulation, thus offering new targets and approaches for cancer treatment.

## 3 Controversies and future prospects

### 3.1 Putative molecular mechanism of tsRNA in regulating PCD

The PCD regulation by tsRNAs involves complex molecular mechanisms. In *Caenorhabditis elegans*, the epidermal growth factor (EGF)-like ligand LIN-3 functions as an external signal to promote PCD in specific cells. This signal is transmitted via the LET-23 receptor, activating the LET-60/RAS–MPK-1/ERK MAPK pathway and the downstream transcription factor LIN-1, which in turn activates the key pro-apoptotic gene *egl-1*, leading to cell death [61]. Although this study did not directly involve tsRNAs, it provides a valuable reference for understanding the regulatory mechanisms underlying PCD.

In cancer research, noncoding RNAs (ncRNAs) play a vital role in regulating PCD. As a subclass of ncRNAs, tsRNAs may contribute to the regulation of PCD through similar mechanisms [62]. For instance, tsRNAs can interact with mRNAs to modulate the expression of related genes, thereby regulating processes such as apoptosis and autophagy. Additionally, tsRNAs may be involved in modulating key molecules in PCD-related signaling pathways, such as indirectly regulating PCD through the expression or activity of proteins such as p53 [63].

### 3.2 Controversies in tsRNA functional research

Despite significant progress in tsRNA research, several controversies remain regarding its functions. The biogenesis of tsRNAs is still not fully understood. Although specific ribonucleases generate tsRNAs by cleaving tRNAs, the precise regulatory mechanisms and influencing factors remain unclear [15]. Furthermore, the functions of tsRNAs vary across various diseases and may differ even within the same disease depending on factors such as cell type and disease stage. This variability makes it challenging to accurately define and understand their roles accurately [14,64]. For instance, different tsRNAs may have opposing effects on tumor cell behaviors, such as proliferation and apoptosis, in breast cancer. Although tsRNA-26576 promotes breast cancer cell growth, other tsRNAs may suppress tumor progression, adding complexity to their functional characterization. Moreover, the interactions between tsRNAs and other ncRNAs, such as miRNAs and lncRNAs, and their cooperative or antagonistic relationships within complex biological networks require further exploration.

tsRNAs also exhibit tissue-specific expression patterns, which complicates their functional analysis. Some studies suggest their role in regulating cell proliferation and apoptosis, whereas others indicate that their functions vary by tissue type. For example, tsRNAs may function differently in cancerous tissues compared with normal tissues, highlighting the need for further investigations into their tissue-specific functions [65].

A major challenge in tsRNA research is the variability and reproducibility of experimental findings. Many studies have reported conflicting results, especially regarding the role of tsRNAs in disease progression. Variations in experimental conditions, such as sample type and processing methods, can lead to inconsistent results, underscoring the need for standardized protocols in tsRNA research [66]. Moreover, the detection and quantification of tsRNAs are significantly influenced by the sequencing platform used. Different high-throughput sequencing technologies, such as RNA sequencing, microarrays, and qPCR, can introduce biases in the detection of specific tsRNA species. Differences in platform sensitivity, resolution, and data analysis methods can lead to inconsistent tsRNA profiles, suggesting that more robust cross-platform validation approaches are needed to ensure accurate characterization of tsRNAs [16].

### 3.3 Future directions in PCD research

PCD research has broad development prospects in the future. Mechanistically, it is essential to investigate the interactions and regulatory networks among various forms of cell death, such as apoptosis, autophagy, and necroptosis, including their signaling crosstalk and cooperative regulation mechanisms, to achieve a more comprehensive understanding of cell death regulation [1]. In cancer treatment, developing more precise and effective therapeutic strategies based on PCD mechanisms is a key direction. As illustrated in Fig. 2, future studies should focus on integrating multiomics data (e.g., transcriptomics and metabolomics) to identify novel tsRNA-targeted therapies. For example, combining PD-1/PD-L1 inhibitors with tsRNA-based immunotherapeutics (e.g., PH-762) can enhance systemic antitumor immunity. Likewise, the development of nanoparticle-based delivery systems for tsRNA mimics or inhibitors (Fig. 2) may help overcome current challenges related to drug specificity and off-target effects. This includes combining the PCD characteristics of tumor cells with individual patient differences to achieve personalized treatment. Additionally, identifying new PCD-related targets and drugs, as well as optimizing current immunotherapy and targeted therapy approaches, can improve cancer treatment outcomes. In other diseases, such as neurodegenerative disorders, understanding the role of PCD in disease progression can lead to new therapeutic approaches, such as slowing disease progression by regulating neuronal PCD.

### 3.4 Challenges and opportunities of tsRNAs in clinical application

The clinical application of tsRNAs present both challenges and opportunities. On the one hand, despite advancements in tsRNA detection technologies, further optimization is still needed to improve accuracy, sensitivity, and convenience. Standardizing these detection methods is essential to facilitate comparisons between different laboratories and clinical applications [11,12]. Drug delivery remains a significant challenge for tsRNAs as therapeutic targets. Developing strategies to efficiently and specifically deliver tsRNA-targeted therapeutic drugs to target cells or tissues, while avoiding off-target effects and adverse reactions, remains a key issue [67].

On the other hand, tsRNAs present numerous opportunities. Their dysregulated expression in various diseases makes them promising candidates as novel biomarkers for early diagnosis, prognostic evaluation, and treatment monitoring, thus providing a basis for precise diagnosis and treatment of diseases [9,19]. Moreover, an in-depth understanding of tsRNA functions and mechanisms and tsRNA-based therapeutic strategies, such as tsRNA-targeted therapy and immunotherapy, may lead to breakthroughs in disease treatment and provide more effective therapeutic options for patients.

## CRediT authorship contribution statement

**Zhe Li:** Writing – review & editing, Writing – original draft. **Bo Zhang:** Visualization. **Yanru Pan:** Validation. **Qiuyan Weng:** Data curation. **Kefeng Hu:** Writing – review & editing.

## Ethics approval and consent to participate

Not applicable.

## Funding

This review was supported by the 10.13039/100007834Ningbo Natural Science Foundation (No. 2024J399), the Ningbo Top Medical and Health Research Program (No.2023020612) and the Ningbo Leading Medical & Healthy Discipline (2022-S04).

## Declaration of competing interest

The authors declare that they have no known competing financial interests or personal relationships that could have appeared to influence the work reported in this paper.

## Abbreviations

tsRNAs:tRNA-derived small RNAs; PCD:Programmed Cell Death; tRFs:tRNA-derived Fragments; tiRNAs:tRNA-derived stress-induced small RNAs; ncRNAs:Non-coding RNAs; CRISPR-Cas:Clustered Regularly Interspaced Short Palindromic Repeats-associated systems; CHA:Catalytic Hairpin Assembly; NGS:Next-Generation Sequencing; RNAi:RNA Interference; SERS:Surface-Enhanced Raman Spectroscopy; ACS:Acute Coronary Syndrome; PRKCA:Protein Kinase C Alpha; FLT1:Fms-Like Tyrosine Kinase 1; PINK1:PTEN Induced Kinase 1; LC3:Microtubule-associated protein 1A/1B-light chain 3; PD-1:Programmed cell death protein 1; PD-L1:Programmed death ligand 1; NLRP3:NLR Family Pyrin Domain Containing 3; GPX4:Glutathione Peroxidase 4; mTOR:Mechanistic Target of Rapamycin.
